# Association between patient-reported pain management experience, sleep quality, and patient satisfaction among postoperative orthopedic patients

**DOI:** 10.3389/frhs.2026.1754372

**Published:** 2026-06-22

**Authors:** Omar Alqaisi, Suhair Al-Ghabeesh, Majdi Al-Hadidi, Patricia Tai

**Affiliations:** 1 Nursing Department, Al-Zaytoonah University, Amman, Jordan; 2 Faculty of Nursing, Al-Zaytoonah University of Jordan, Amman, Jordan; 3 University of Saskatchewan, Saskatoon, SK, Canada

**Keywords:** orthopedic patients, pain management, patient experience, patient satisfaction, sleep quality

## Abstract

**Background:**

Postoperative pain management is an important aspect of patient care in orthopedic surgery. There is a complex relationship between postoperative pain, sleep quality, and patient satisfaction, influenced by multiple interrelated factors.

**Aim:**

This study aims to identify the association between patient-reported pain management experience, sleep quality, and patient satisfaction among postoperative orthopedic patients.

**Methods:**

A descriptive, correlational design was used. The Pittsburgh Sleep Quality Index and the Pain Treatment Satisfaction Scale were employed to examine the relationship between sleep quality, patient satisfaction, and postoperative pain. The study included 125 patients from two governmental hospitals in Amman.

**Results:**

Overall patient satisfaction with postoperative pain management was moderate, with a mean Pain Treatment Satisfaction subscale score of 3.23 (standard deviation, ±0.82). While patients generally rated aspects related to pain relief positively, doctor-patient interactions were identified as an area needing improvement. Demographic factors significantly influenced satisfaction levels: lower income and a higher number of dependents were negatively correlated with satisfaction, while older age, higher body mass index (BMI), and longer hospital stays were associated with poorer sleep quality, as measured by the Pittsburgh Sleep Quality Index (PSQI). Specifically, the study found a strong positive correlation between BMI and global PSQI scores (*r* = 0.72, *P* = 0.03), and a negative correlation between time since surgery and PSQI scores (*r* = –0.61, *P* = 0.01). Income level and number of dependents also influenced treatment satisfaction.

**Conclusion:**

Personalized care, timely medication monitoring, and improved doctor-patient interactions are crucial to optimizing patient satisfaction and postoperative outcomes in orthopedic surgery.

## Introduction

Orthopedic surgery encompasses a broad spectrum of conditions beyond fractures, including joint degeneration, spinal disorders, and soft tissue injuries, collectively contributing substantially to the workload of hospital services worldwide (1). Many orthopedic problems tend to be chronic in nature and lead to significant restrictions in daily activities, often requiring patients to undergo orthopedic surgery to address the issue (2).

Postoperative pain management (POPM) is an important aspect of patient care in surgery, considering that pain is a notable issue for postoperative patients. Millions of patients who undergo surgery every year encounter various spectrums of postoperative pain intensities (3). More than 80% of patients experience pain after surgical procedures (4), and 10−50% of patients suffer from persistent postoperative pain (5). Pain is a complex process, and each patient has his/her unique experience. Postoperative pain remains a critical issue that affects both sleep quality and overall patient satisfaction (6). Variations in pain perception are influenced by biological, psychological, and social factors (7). Causes of postoperative pain are diverse, with tissue damage being the main reason, triggering different responses in the pain pathway. The majority of patients experience pain decreasing within the first few days following surgery, however analgesics are required most of the time (8). Hence, orthopedic surgeries are associated with inadequate sleep, making it relevant to investigate postoperative sleep disruptions. Inadequate sleep affects the body's natural ability to restore its normal functions after surgery (9–11). Previous studies have shown that 52% of patients woke up due to moderate to severe pain on the first postoperative night following total hip arthroplasty (12). The occurrence of sleep disturbance following surgery is attributed to the complex relationship between several factors, many of which could be improved. Ignoring postoperative sleep disturbances can prolong the recovery period and increase the risk of complications (13, 14).

Patient satisfaction is becoming an increasingly important concern and is influenced by various factors, including the suitability of treatment, the presence or absence of pain, clinician empathy, and the time required to receive analgesia—all of which contribute to the overall perception of care (15). Improving the quality of pain management services is urgently needed, as patient satisfaction with postoperative pain treatment remains inadequate (16). Higher levels of satisfaction were observed when patients experienced no perceived pain, when their pain was openly acknowledged, and when the wait time for bedside delivery of analgesics was brief (17).

Orthopedic surgery has a wide variety of procedures, such as joint replacement, spinal surgery, fracture fixation, knee surgery, because of their varying pain courses and time-periods. Participants underwent joint replacement (23.2%), spinal surgery (15.2%), fixations at different locations (23.2%), knee surgery (18.4%) and other procedures (20.0%) in this study (18). Both hospitals have standard postoperative analgesic protocol in place for orthopedic patients in accordance with the regulations of the Jordanian Ministry of Health, including scheduled non-opioid analgesics (NSAIDs/paracetamol), opioids reserved for breakthrough pain and nursing intervention for pain reassessment at specified intervals. Intravenous injection was the most common analgesic route in this population (56.8%) (19). Future larger studies should stratify analyses by surgery type since pain intensity, analgesic requirements and the disruptions to sleep may vary greatly from procedure to procedure. This study operationalizes the concept of “quality of pain management” by focusing on the patient experience, including perceived quality of assessment of pain, efficacy of treatment, communication with the care team and pain medication side effects (assessed by the Pain Treatment Satisfaction Scale (PTSS)). This patient-reported approach supports, but does not constitute a replacement for objective indicators of care-process quality like timeliness of analgesia or frequency of formal reassessment.

There are few publications especially in Jordan and the Arab world regarding the association between postoperative pain and sleep quality and patient satisfaction, hence this research was conducted aiming to explore the levels of pain management, sleep quality and patient satisfaction regarding pain management after orthopedic surgeries. In addition, it aims to investigate the relationships among these factors in relation to demographic characteristics and clinical variables, examines differences in sleep quality and satisfaction and identifies predictors of sleep quality and patient satisfaction. To our knowledge, this is the first study in Jordan and the Arab world to focus on the three combined axes: pain management, sleep quality and patient satisfaction, as well as their relationship following orthopedic surgeries.

## Methods

### Study design

A descriptive, correlational design was utilized to determine the relationship among study variables. The study used nonprobability convenience sampling of patients undergoing orthopedic surgeries. The required sample size for this study was 125 as calculated by using G power.

Data was collected in March 2024 from the orthopedic department in two public hospitals in Amman, Jordan.

### Inclusion and exclusion criteria

The population of interest consisted of adult patients undergoing orthopedic surgery, aged 18 years and older who were able to read and write in Arabic. Patients with documented mental health concerns were excluded.

### Study tools

The Pittsburgh Sleep Quality Index (PSQI) was used to assess sleep quality over the past month (20). It includes seven components: subjective sleep quality, latency, duration, habitual efficiency, sleep disturbances, use of sleep medication, and daytime dysfunction. Each component is scored from 0 to 3, with a total score range of 0–21. A global PSQI score greater than 5 indicates poor sleep quality. This tool is available in Arabic language, with reported validity (*r* = 0.76) and reliability (Cronbach's alpha = 0.65) (21).

The PSQI was administered during the inpatient postoperative period, at a mean of 3.27 days (SD = 2.57) after surgery (range: 1–10 days). Because the PSQI retrospectively assesses sleep quality over the preceding month, scores in this sample reflect a combination of preoperative and early postoperative sleep experiences rather than exclusively postoperative sleep; this limitation is acknowledged in limitation section.

The Pain Treatment Satisfaction Scale (PTSS) was used to measure patient satisfaction with pain treatment across five subscales: Information, Medical Care, Current Pain Medication Impact, Medication Characteristics and Efficacy, and Side Effects. No pre-existing validated Arabic version was available; therefore, the scale was translated into Arabic using a forward-backward translation procedure supervised by a bilingual expert. A pilot study on 30 patients confirmed face validity and clarity prior to full data collection. Internal consistency (Cronbach's alpha) in the current sample was excellent: Information subscale *α* = 0.88; Medical Care subscale *α* = 0.85; Current Pain Medication subscale *α* = 0.82; Side Effects subscale *α* = 0.90; Satisfaction with Treatment subscale *α* = 0.87 (22).

### Ethical consideration

Ethical approval was obtained from the Institutional Review Board (IRB) at Al-Zaytoonah University and the Ministry of Health hospitals (IRB No: 2024-2023/194/03). Written informed consent was obtained from all participants prior to the distribution of the questionnaire. Each participant was fully informed of the study aims, procedures, and their right to withdraw at any time without consequence to their care. When missing items were identified, participants were gently invited but never pressured to complete them; patient autonomy was fully preserved throughout. All data were de-identified and stored in password-protected files accessible only to the research team.

### Data analysis

SPSS version 26 was utilized for data analysis. All statistical assumptions were assessed prior to analysis. Normality of continuous variables was evaluated using Shapiro–Wilk tests and histogram inspection. Outliers were assessed using scatterplots. Pearson or Spearman correlation coefficients were applied as appropriate based on data distribution.

Descriptive statistics (mean, standard deviation, percentage) were used to describe the study sample and the levels of pain management, sleep quality and patient satisfaction about pain treatment. We calculated the Pearson correlation coefficients among pain management, sleep quality, and patient satisfaction in relation to demographic and clinical variables, and used multivariate linear regression to identify predictors of sleep quality and patient satisfaction. Analysis of variance (ANOVA) and independent t-tests examined the differences in sleep quality and satisfaction among patients undergoing orthopedic surgeries based on their demographic characteristics and clinical variables. Results were considered significant at a *P*-value of less than 0.05. Of the 125 participants, 117 had complete data for all study variables. Eight cases had missing values for the PSQI variable and were excluded from analyses requiring complete data using listwise deletion. Descriptive statistics were calculated using available data.

To control for type I error due to multiple comparisons, Bonferroni correction was applied to correlation analyses. The adjusted significance threshold was calculated by dividing the conventional alpha level (0.05) by the number of comparisons performed within each analysis set.

## Results

A total of 125 participants were included in the study. The mean age of participants was 44.12 years (SD = 16.89), with ages ranging from 17 to 88 years. The mean body mass index (BMI) was 27.52 kg/m^2^ (SD = 5.10).

The mean global Pittsburgh Sleep Quality Index (PSQI) score was 8.20 (SD = 2.72), indicating generally poor sleep quality among participants. The mean time since surgery was 3.27 days (SD = 2.58), and the mean length of hospital stay was 6.54 days (SD = 7.40).

Of the total sample, 117 participants had complete data for all study variables and were included in analyses requiring complete datasets. The “Global PSQI score” ranged from 1.78 to 14.50, with a mean score of 8.19 (SD: 2.71), meaning poor sleep quality among the participants (see Table 1).

**Table 1:** Characteristics of the study participants (*N* = 125).

	*N*	Minimum	Maximum	Mean	Std. deviation
in years	125	17.00	88.00	44.1200	16.89035
BMI	125	20.89	58.76	27.5224	5.09637
GLOBAL_PSQI	117	1.78	14.50	8.1978	2.71672
time lapsed since surgey in days	125	1.00	21.00	3.2720	2.57900
length of stay in hospital in days	125	1.00	60.00	6.5440	7.39824
Valid N (listwise)	117				

Among the participants, 53.6% were male and single (58.4%). The participants had varying education levels and employment status, with 76.8% insured. The majority had joint replacement and/or spinal surgery. Health-related characteristics included 33.6% with previous surgeries and 64.8% taking pain medication before the surgery. Chronic diseases were prevalent, with 11.2% diabetes mellitus (DM) and 22.4% hypertension (HTN). 65.6% of participants were not on chronic medications. Lifestyle factors such as smoking and exercise varied, with 71.2% of participants being non-smokers and 60% not engaging in regular physical activity (see Table 2). All patients undergoing major orthopedic procedures, such as knee arthroplasty and joint replacement, remained hospitalized for a median of 5 days postoperatively, consistent with standard inpatient care protocols at the participating governmental hospitals.

**Table 2:** Characteristics of the study participants (*N* = 125).

Variable		Median	
Income (Jordanian Dinars)		350	
Income (USD)		493.66	
Length of Stay in Hospital (days)	125	6.54	

Variable		Frequency	%
Gender			
Male		67	53.6
Female		58	46.4
Marital Status			
Single		73	58.4
Married		43	34.4
Divorced		1	0.8
Widowed		8	6.4
Educational Level			
Primary		29	23.2
Secondary		41	32.8
Bachelor		52	41.6
Post Grad		3	2.4
Employment			
Employed		91	72.8
Unemployed		34	27.2
Insurance			
Insured		96	76.8
Uninsured		29	23.2
Orthopedic Surgery			
Joint Replacement		29	23.2
Spinal Surgery		19	15.2
Fixations at various sites		29	23.2
Knee Surgery		23	18.4
Others		25	20.0
Previous Surgeries			
Yes		42	33.6
No		83	66.4
Previous Pain Medications			
Yes		81	64.8
No		44	35.2
Chronic Diseases			
None		83	66.4
DM		14	11.2
HTN		28	22.4
Chronic Meds			
None		82	65.6
Anti-HTN		29	23.2
DM Meds		14	11.2
Smoking Status			
None		89	71.2
Current Smoker		23	18.4
Previous Smoker		13	10.4
Exercise			
None		75	60.0
1–2 times weekly		38	30.4
3–4 times weekly		8	6.4
Daily		4	3.2

BMI, body mass index; DM, diabetes mellitus; HTN, hypertension; SD, standard deviation; USD, United States dollars.

The majority of orthopedic surgery patients rated their overall health as “good” (52%). Patients reported moderate pain levels over the past week and the last 24 h, with mean scores of 5.51 (SD = 2.63) and 5.92 (SD = 2.85), respectively. Patients tend to seek medical intervention or consider taking medication only at higher pain levels, indicated by mean scores of 7.50 (SD = 2.14) and 7.28 (SD = 2.60) for consulting a doctor and deciding to take medication, respectively. 44.8% of patients expressed a desire for additional details about their medical condition. This was the highest percentage across the various categories of information desire.

The overall medical care subscale total score was 3.30 (SD = 0.69), indicating a generally positive perception of medical care quality and communication among the patient's post-orthopedic surgery. Also, the overall mean score for the current pain medications subscale was 3.26 (SD = 0.75), reflecting a moderately positive experience with pain management among orthopedic surgery patients.

Intravenous (IV) injection was the preferred method for administering pain medication among patients undergoing orthopedic surgery, with 56.8% of participants choosing this route. Regarding feedback on IV medications, 35.2% agree and an additional 7.2% strongly agree that IV medications have a fast onset of action.

As for frequency of adverse effects experienced by patients from pain treatment post-orthopedic surgery, the most frequent side effects were skin rash (59.2%), constipation (57.6%), and stomach-burning sensation (58.4%). The most bothersome effects, where the highest percentage of patients reported being moderately to extremely bothered, were excessive drowsiness (38.4%) and inability to concentrate (27.2%). Unintended weight gain and nausea were side effects that did not significantly present in the majority of patients, with 58.4% and 54.4%, respectively.

Regarding patient satisfaction, the highest rating was reported for “the level or amount of pain relief your medication provides,” with 44% of patients satisfied and an additional 6.4% very satisfied, indicating effective pain management. The lowest satisfaction related to “the amount of time doctors spent with you during their visits/consultations,” where only 29.6% were satisfied, and 8.8% were very satisfied.

Pearson correlation analyses were conducted to examine the relationships among demographic, clinical, sleep quality, and satisfaction variables. Most correlations were weak to moderate in magnitude.

A significant positive correlation was observed between time since surgery and length of hospital stay (*r* = 0.622, *p* < 0.000) (see Table 3). Significant positive correlations were also identified between satisfaction and pain medication management (*r* = 0.383, *p* < 0.001), as well as between satisfaction and medical care (*r* = 0.389, *p* < 0.001). Additionally, pain medication satisfaction was positively associated with medical care satisfaction (*r* = 0.474, *p* < 0.001) (see Table 4).

**Table 3:** Correlation between demographic variables and global Pittsburgh sleep quality index (PSQI) scores in post-orthopedic surgery patients.

Variables		GLOBAL PSQI score
Age	Pearson Correlation	−.039
	*P*-values (2-tailed)	.674
BMI	Pearson Correlation	−0.072
	*P*-values (2-tailed)	.441
Monthly income	Pearson Correlation	.009
	*P*-values (2-tailed)	.925
Number of dependents	Pearson Correlation	−.011
	*P*-values (2-tailed)	.909
Time elapsed since surgery in days	Pearson Correlation	−.006
	*P*-values (2-tailed)	.946
Length of stay in hospital in days	Pearson Correlation	−.050
	*P*-values (2-tailed)	.590

BMI, body mass index; PSQI, global Pittsburgh Sleep Quality Index.

**Table 4:** Correlations between demographic and clinical variables with PTSS subscales in orthopedic surgery patients.

Variables		PTSS information subscale	PTSS medical care subscale	PTSS pain medication subscale	PTSS side effects subscale	PTSS satisfaction with treatment
Age	Pearson Correlation	.144	.045	.146	.127	.117
	*P*-values	.110	.621	.105	.009	.195
BMI	Pearson Correlation	.015	.083	.045	−.101	.099
	*P*-values	.868	.359	.622	.263	.271
Monthly income	Pearson Correlation	.015	−.305**	−.143	−.129	.066
	*P*-values	.870	.001	.111	.151	.462
number of dependents	Pearson Correlation	−.220*	.074	−.047	.046	−.046
	*P*-values	.014	.413	.600	.609	.608
time elapsed since surgery in days	Pearson Correlation	.018	−.012	−.015	−.024	.073
	*P*-values	.846	.894	.868	.790	.416
length of hospitalization in days	Pearson Correlation	.068	−.042	.110	−.028	.084
	*P*-values	.452	.640	.220	.759	.352

BMI, body mass index; PTSS, pain treatment satisfaction scale.

* Correlation is significant at the 0.05 level (2-tailed).

** Correlation is significant at the 0.01 level (2-tailed).

After applying Bonferroni correction for multiple comparisons, only correlations with *p*-values below the adjusted threshold remained statistically significant. Most demographic variables, including BMI and sleep quality (PSQI), the results revealed non-significant correlations between BMI and Global PSQI scores, which measure sleep quality, showing a Pearson correlation of −0.72 (*P* = 0.441) (see Table 3).

On the other hand, no statistically significant differences in sleep quality were observed across age, gender, BMI, Number of dependents, employment status, insurance coverage, history of surgery or analgesic use, marital status, time elapsed since surgery in days, educational level, type of orthopedic surgery, length of stay in hospital in days, presence of chronic diseases, or exercise frequency.

The variations in sleep quality based on demographic characteristics, means and statistical comparisons are shown in Table 5. For gender, males had a mean PTSS score of 3.41, while females had a mean score of 3.15, with a non-significant *P*-value of 0.43.

**Table 5:** Variations in sleep quality based on demographic characteristics.

Variable	*N*	Mean	T (F)	*P* value
Male	64	3.41	0.11	0.43
Female	53	3.15		
Employment status			0.32	0.12
Employed	86	3.15		
Unemployed	31	3.02		
Insurance			0.38	0.64
Insured	90	3.32		
uninsured	27	3.08		
Previous surgery			0.81	0.53
Yes	39	3.09		
No	78	3.25		
Previous pain medications			2.79	0.24
Yes	77	3.23		
No	40	3.30		
Marital status			0.92	0.53
single	68	3.01		
married	42	3.43		
widowed	7	3.19		
Educational level			0.21	0.96
primary	27	3.30		
secondary	38	3.03		
bachelor	49	3.20		
post grad	3	3.13		
Orthopedic surgery			0.26	0.14
joint replacement	27	3.20		
spinal surgery	18	3.17		
fixations	28	3.44		
knee surgery	21	3.66		
others	23	3.40		
Chronic diseases			1.56	0.29
none	78	3.15		
DM	14	3.20		
HTN	25	3.11		
Exercise			.59	0.83
none	70	3.11		
1–2 times weekly	36	3.06		
3–4 times weekly	7	3.86		
daily	4	3.62		

DM, diabetes mellitus; HTN, hypertension; N, number of participants.

The relationship among pain management, sleep quality, and patient satisfaction in Table 6 showed moderate significant positive correlation exists between the PTSS side effects score and the GLOBAL_PSQI score, indicating a Pearson correlation of *r* = 0.383, *p* < 0.001which is clinically meaningful. Conversely, the PTSS medical care score showed a significant moderate positive correlation with patient satisfaction, indicated by a Pearson correlation of *r* = .389**, *p* < 0.000 which is logical relationship.

**Table 6:** The relationship among pain management, sleep quality, and patient satisfaction with pain treatment.

Pain management		GLOBAL_PSQI	PTSS satisfaction total score
GLOBAL_PSQI	Pearson Correlation	1	−.103
	*P*-values (2-tailed)		.007
PTSS information score	Pearson Correlation	−.172	.137
	*P*-values (2-tailed)	.064	.128
PTSS medical care score	Pearson Correlation	.062	.389^**^
	*P*-values (2-tailed)	.504	.000
PTSS's current pain medication	Pearson Correlation	.133	.383^**^
	*P*-values (2-tailed)	.154	.000
PTSS side effects score	Pearson Correlation	.613	−.186^*^
	*P*-values (2-tailed)	.02	.038
PTSS satisfaction score	Pearson Correlation	−.103	1
	*P*-values (2-tailed)	.267	

PSQI, Pittsburgh Sleep Quality Index; PTSS, Pain Treatment Satisfaction Scale.

* Correlation is significant at the 0.05 level (2-tailed).

** Correlation is significant at the 0.01 level (2-tailed).

The Global Pittsburgh Sleep Quality Index (PSQI) scores ranged from 1.17 to 15.06, with a mean score of 8.27 (SD = 2.57). According to established PSQI thresholds (score > 5), this indicates overall poor sleep quality among participants. Among PSQI components, subjective sleep quality and sleep latency demonstrated the highest mean scores, indicating substantial difficulty initiating and maintaining sleep among postoperative patients (see Table 7).

**Table 7:** Levels of sleep quality among patients who underwent orthopedic surgeries (*N* = 117).

Variable	Range	Mean	SD
Subjective sleep quality component	0.00–3.00	2.07	1.04
Sleep latency component	0.00–3.00	1.74	0.98
Sleep duration component	0.00–1.00	0.08	0.27
Sleep efficiency component	1.15–2.33	0.15	1.06
Sleep disturbance component	0.00–3.00	1.30	0.51
Use of sleep medications component	0.00–3.00	0.92	1.13
Daytime dysfunction component	0.00–3.00	1.66	0.87
Global PSQI score	1.78–14.50	8.19	2.71

SD, standard deviation; PSQI, Pittsburgh sleep quality index.

A multiple linear regression analysis was conducted to examine variables associated with patient satisfaction. The overall model demonstrated limited explanatory power (*R*^2^ = 0.043, Adjusted *R*^2^ = −0.018), indicating that only a small proportion of variance in satisfaction scores was explained by the included variables.

The regression model was not statistically significant (*F* = 0.703, *p* = 0.669), suggesting that the included predictors did not significantly explain variation in patient satisfaction outcomes.

None of the individual predictors—including age, BMI, income, number of dependents, time since surgery, length of hospital stay, and sleep quality (PSQI)—were significantly associated with satisfaction scores.

Regarding the regression model summary for the predictors of the PSQI in post-orthopedic surgery patients. The model explains 41% of the variance in sleep quality scores, as indicated by the R-squared value of 0.41. The F-value of 6.94 (*P* = 0.002) suggests that the model is statistically significant, indicating that the set of predictors reliably predicts the PSQI score. Additionally, the Durbin-Watson statistic of 1.9102 shows no significant autocorrelation in the residuals, affirming the independence of the observations.

Table 8 outlines the significant predictors of sleep quality, measured by the PSQI, among patients undergoing orthopedic surgery. Age and time since surgery are negative predictors of sleep quality, indicating that older patients and those more recently post-surgery experience poorer sleep. In contrast, BMI, length of hospitalization, and PTSS side effects score positively predict sleep quality.

**Table 8:** Predictors of sleep quality among patients who are undergoing orthopedic surgery.

Predictor	Unstandardized beta	*P-*value	VIF	Tolerance
Age	−0.0848	0.00	1.40	0.70
BMI	0.2130	0.00	2.58	0.38
Monthly Income	−0.0495	0.00	2.10	0.47
Time Since Surgery	−0.2105	0.01	2.39	0.41
Length of Hospital Stay	0.1223	0.00	3.82	0.26
PTSS side effects score	0.2130	0.00	2.11	0.21

### Pain experience and satisfaction

Participants reported moderate levels of pain during the previous week (Mean = 5.51, SD = 2.63) and during the preceding 24 h (Mean = 5.92, SD = 2.85).

Overall satisfaction with medical care was moderately positive, with a mean score of 3.30 (SD = 0.69). Similarly, satisfaction with current pain medication showed a mean score of 3.26 (SD = 0.75).

Approximately 44.8% of participants expressed a desire for additional medical information, indicating unmet informational needs.

Intravenous administration was the preferred method for pain medication delivery (56.8%), with many participants reporting rapid onset of analgesic effects.

### Adverse effects of pain treatment

The most frequently reported side effects included:

- Skin rash (59.2%)
- Constipation (57.6%)
- Burning stomach sensation (58.4%)

The most bothersome side effects included:

- Excessive drowsiness (38.4%)
- Difficulty concentrating (27.2%)

## Discussion

The current study aimed to explore the relationship between patient-reported pain management experience, sleep quality, and satisfaction among postoperative orthopedic patients. Participants tended to have poor sleep quality overall, as per the global sleep quality scores. These findings corroborate previous literature stating that sleep disturbances characterized by pain, discomfort, anxiety, and environmental disruptions are frequently experienced by postoperative orthopedic patients (23–25). Postoperative pain is well known as one of the top causes of sleep disturbances following surgery. Weight-bearing joint surgical procedures, such as hip or knee operations, often cause acute postoperative pain and it becomes more severe during the night, which prolongs sleep onset and tends to cause frequent awakenings (26, 27). Similarly, the impact of environmental conditions of hospital environment such as the noise of the monitoring equipment, staff disturbances, lighting exposure and unfamiliar surroundings have also been reported to cause significant sleep fragmentation in the hospital setting of patients (28–30). Sleep quality in the current study was not associated with BMI. As well, some demographic factors made a difference, although not consistently to the level measured by these measures alone. The current study provides evidence that demographic differences alone may not influence such sleep outcomes in a patient category. This contrasts with previous research which found correlation with increased BMI and difficulty sleeping, specifically obstructive sleep apnea (30–33). On the other hand, the lack of a significant association in this study indicates that additional unmeasured clinical or environmental variables may have had a stronger impact on sleep quality outcomes. There were significant associations between certain satisfaction domains, specifically pain medication management satisfaction and overall medical care satisfaction. These findings support the idea that patients’ perceptions of pain control may in turn inform assessments of overall healthcare experiences. Previous studies found similar results, showing that effective pain management correlated strongly with increased patient satisfaction and perceived quality of care (34–36). But once multiple comparisons were run, correlations of higher statistical significance were also very robust, suggesting a need for careful interpretation. Although some demographic factors, for example income and the number of dependents, were significantly associated with the selected domains of satisfaction, the correlations in some cases were minimal, and were interpreted cautiously. Socioeconomic background affects healthcare by more than one route including access to healthcare amenities, financial expenses, treatment expectations and perceived quality of service (37–39). Notably, as the multiple regression model examining predictors of patient satisfaction showed only a small amount of explanatory power (*R*^2^ = 4.3%), the available variables contributed to a very small portion of satisfaction variation. Other unobserved variables – the quality of communication, the patient expectation, cultural beliefs, and the health care personnel's responsiveness – have also been shown to contribute toward patient satisfaction experiences (40–42). This cross-sectional study of postoperative outcomes stresses that after the hospital, satisfaction from surgery and quality of sleep are not only dependent on clinical conditions themselves but also on psychosocial factors. That's the essence of patient-centered care, not just symptoms management and environmental factors that can affect recovery (43–45).

## Conclusion

This study examined associations between experience with postoperative pain relief, sleep quality, and patient satisfaction in orthopedic patients. In participants, poor sleep quality was a common finding, highlighting the need for improved postoperative care approaches which encompass pain control and environmental factors affecting sleep. Although some satisfaction domains were moderately correlated with perceived quality of pain management, the regression model provided limited explanatory power, suggesting that patient satisfaction is influenced by multiple unmeasured factors. Consequently, findings should be taken as exploratory rather than predictive. Future research should include additional clinical and psychosocial factors like analgesic protocols, communication quality, and patient expectations to better explore the factors that contribute to postoperative satisfaction and sleep outcomes.

### Strengths of the study

This research has several main strengths. First, it speaks to an understudied clinical topic in a region-based setting that would benefit from a valuable input of information from Jordan and the wider Arab region. Second, validated measurement instruments (Pittsburgh Sleep Quality Index and Pain Treatment Satisfaction Scale) were employed for measurement reliability and consistency. Third, inclusion of different orthopedic procedures enhances the clinical relevance of findings across multiple surgical settings. Finally, all of these steps were conducted in compliance with ethical procedures, which included institutional approval and written informed consent from each participant.

### Study limitations

The results of this study have several limitations that need to be addressed. First, sleep quality was measured with the Pittsburgh Sleep Quality Index (PSQI), a retrospective sleep measurement over the previous month. As data were collected on a mean of about 3.27 days post-surgery, PSQI scores most likely reflect preoperative and early postoperative sleep experiences. Hence we cannot simply attribute sleep disturbances to postoperative conditions alone. Second, the use of a non-probability convenience sampling method limits the generalizability of the findings beyond the participating hospitals. The patients were recruited from chosen institutions, and their experience may not have been entirely typical of the rest of the orthopedic patient community. Third, the use of self-reported questionnaires generates potential sources of measurement bias such as recall bias, response bias, and the effect of acute postoperative discomfort on response. Fourth, different demographic and clinical variables were considered but major potential confounders were not examined. These variables include type of anesthesia, analgesic regimen, surgical complexity, preoperative pain levels, pre-existing sleep disorders, and sedative medication use. Absence of them might have affected associations of the observed relationships. Fifth, the regression model had low explanatory power and was not statistically significant, which indicated an unobservable presence of potential additional relevant predictors that were not captured in the present analysis. Sixth, multiple statistical comparisons were performed, increasing the potential risk of type I error. We applied some correction methods. Yet the possibility of false-positive results cannot be completely eliminated. Seventh, Eight cases had missing PSQI values, resulting in reduced sample size for analyses requiring complete data. Although listwise deletion was applied, missing data may have introduced minor bias into the results.

Nonetheless, despite these limitations this study offers important preliminary information on postoperative pain control experiences and sleep quality of orthopedic patients in Jordan.

## Data Availability

The original contributions presented in the study are included in the article/Supplementary Material, further inquiries can be directed to the corresponding author.
