# Supplementary material for: Association between patient-reported pain management experience, sleep quality, and patient satisfaction among postoperative orthopedic patients
Source: Front Health Serv. 2026 Jun 22;6:1754372. doi: 10.3389/frhs.2026.1754372 (PMC13333413; doi:10.3389/frhs.2026.1754372)
Supplement: Supplementary file 1 [file Table1.docx]

**Appendix A.1**

Appendix 1: Patient Preferences on the Provision of Information Regarding Pain Management and Treatment Options Post-Orthopedic Surgery

| **Treatment information questions** | **Frequency** | **Percent** |
| --- | --- | --- |
| **My Medical Condition or Injury** |  |  |
| I would like to get more information | 56 | 44.8 |
| I would like a little more information | 21 | 16.8 |
| The amount of information provided is suitable for me | 43 | 34.4 |
| I would prefer to have less information | 5 | 4.0 |
| **The Causes of the Pain I am Experiencing** |  |  |
| I would like to get more information | 42 | 33.6 |
| I would like a little more information | 32 | 25.6 |
| The amount of information provided is suitable for me | 38 | 30.4 |
| I would prefer to have less information | 13 | 10.4 |
| **Treatment Options for My Pain** |  |  |
| I would like to get more information | 48 | 38.4 |
| I would like a little more information | 39 | 31.2 |
| The amount of information provided is suitable for me | 27 | 21.6 |
| I would prefer to have less information | 11 | 8.8 |
| **Pain Medications, in General** |  |  |
| I would like to get more information | 50 | 40.0 |
| I would like a little more information | 31 | 24.8 |
| The amount of information provided is suitable for me | 34 | 27.2 |
| I would prefer to have less information | 10 | 8.0 |
| **Possible Side Effects of Pain Medications** |  |  |
| I would like to get more information | 53 | 42.4 |
| I would like a little more information | 34 | 27.2 |
| The amount of information provided is suitable for me | 26 | 20.8 |
| I would prefer to have less information | 12 | 9.6 |
| **Variable** | **M** | **SD** |
| **Total score of treatment information subscale** | 2.03 | 0.72 |

M, mean; SD, standard deviation.

**AppendixA.2:** Patient Perceptions of Medical Care Quality and Communication Post-Orthopedic Surgery

| **Medical care subscale questions** | **Frequency** | **Percent** |
| --- | --- | --- |
| **It is easy to ask questions to the medical team** |  |  |
| Strongly Disagree | 8 | 6.4 |
| Disagree | 15 | 12.0 |
| Neutral | 21 | 16.8 |
| Agree | 65 | 52.0 |
| Strongly Agree | 16 | 12.8 |
| **The medical team always does its best to alleviate my concerns** |  |  |
| Strongly Disagree | 5 | 4.0 |
| Disagree | 16 | 12.8 |
| Neutral | 26 | 20.8 |
| Agree | 65 | 52.0 |
| Strongly Agree | 13 | 10.4 |
| **The medical team is willing to provide pain medications that I feel I need** |  |  |
| Strongly Disagree | 7 | 5.6 |
| Disagree | 14 | 11.2 |
| Neutral | 25 | 20.0 |
| Agree | 61 | 48.8 |
| Strongly Agree | 18 | 14.4 |
| **The medical team provides adequate follow-up care** |  |  |
| Strongly Disagree | 10 | 8.0 |
| Disagree | 17 | 13.6 |
| Neutral | 19 | 15.2 |
| Agree | 61 | 48.8 |
| Strongly Agree | 18 | 14.4 |
| **The medical team does not ask me about the pain I am experiencing** |  |  |
| Strongly Disagree | 35 | 28.0 |
| Disagree | 37 | 29.6 |
| Neutral | 19 | 15.2 |
| Agree | 32 | 25.6 |
| Strongly Agree | 2 | 1.6 |
| **Variable** | **M** | **SD** |
| **Medical care subscale total score** | 3.30 | 0.69 |

M, mean; SD, standard deviation.

**Appendix A. 3:** Patient Experience and Perceptions of Current Pain Medication Efficacy Post-Orthopedic Surgery

| **Current pain medications subscale** | **Frequency** | **Percent** |
| --- | --- | --- |
| **The pain medication I take has a positive impact on my physical health** |  |  |
| Strongly Disagree | 15 | 12.0 |
| Disagree | 32 | 25.6 |
| Neutral | 33 | 26.4 |
| Agree | 40 | 32.0 |
| Strongly Agree | 5 | 4.0 |
| **The pain medication I take helps me look at life with greater optimism** |  |  |
| Strongly Disagree | 6 | 4.8 |
| Disagree | 16 | 12.8 |
| Neutral | 38 | 30.4 |
| Agree | 56 | 44.8 |
| Strongly Agree | 9 | 7.2 |
| **The pain medication allows me to perform my daily activities more easily** |  |  |
| Strongly Disagree | 10 | 8.0 |
| Disagree | 14 | 11.2 |
| Neutral | 29 | 23.2 |
| Agree | 62 | 49.6 |
| Strongly Agree | 10 | 8.0 |
| **The pain medication allows me to participate in recreational activities more** |  |  |
| Strongly Disagree | 15 | 12.0 |
| Disagree | 22 | 17.6 |
| Neutral | 36 | 28.8 |
| Agree | 40 | 32.0 |
| Strongly Agree | 12 | 9.6 |
| **The pain medication helps me do things independently** |  |  |
| Strongly Disagree | 9 | 7.2 |
| Disagree | 22 | 17.6 |
| Neutral | 33 | 26.4 |
| Agree | 54 | 43.2 |
| Strongly Agree | 7 | 5.6 |
| **The pain medication allows me to communicate better with others** |  |  |
| Strongly Disagree | 7 | 5.6 |
| Disagree | 18 | 14.4 |
| Neutral | 23 | 18.4 |
| Agree | 68 | 54.4 |
| Strongly Agree | 9 | 7.2 |
| **The pain medication improves my mood** |  |  |
| Strongly Disagree | 9 | 7.2 |
| Disagree | 17 | 13.6 |
| Neutral | 35 | 28.0 |
| Agree | 54 | 43.2 |
| Strongly Agree | 10 | 8.0 |
| **The pain medication helps me concentrate better** |  |  |
| Strongly Disagree | 4 | 3.2 |
| Disagree | 23 | 18.4 |
| Neutral | 33 | 26.4 |
| Agree | 53 | 42.4 |
| Strongly Agree | 12 | 9.6 |
| **Variable** | **M** | **SD** |
| Current pain medications subscale | 3.26 | 0.75 |

M, mean; SD, standard deviation.

**Appendix A.4:** Patient Experiences with Oral Medication Administration Post-Orthopedic Surgery

| **Oral Medications Routes Questions** | **Frequency** | **Percent** |
| --- | --- | --- |
| **The medicines are easy to swallow** |  |  |
| Strongly Disagree | 5 | 4.0 |
| Disagree | 8 | 6.4 |
| Neutral | 4 | 3.2 |
| Agree | 25 | 20.0 |
| Strongly Agree | 8 | 6.4 |
| **The medications leave a taste after swallowing** |  |  |
| Strongly Disagree | 3 | 2.4 |
| Disagree | 5 | 4.0 |
| Neutral | 23 | 18.4 |
| Agree | 14 | 11.2 |
| Strongly Agree | 5 | 4.0 |

**Appendix A.5:** Patient Feedback on Intravenous Medication Administration Experiences Post-Orthopedic Surgery

| **Intravenous Medications Routes Questions** | **Frequency** | **Percent** |
| --- | --- | --- |
| **The meds have a fast onset** |  |  |
| Strongly Disagree | 4 | 3.2 |
| Disagree | 1 | 0.8 |
| Neutral | 17 | 13.6 |
| Agree | 44 | 35.2 |
| Strongly Agree | 9 | 7.2 |
| **The meds cause pain upon administration** |  |  |
| Strongly Disagree | 8 | 6.4 |
| Disagree | 17 | 13.6 |
| Neutral | 23 | 18.4 |
| Agree | 27 | 21.6 |
| **Injections cause bruising** |  |  |
| Strongly Disagree | 10 | 8.0 |
| Disagree | 14 | 11.2 |
| Neutral | 21 | 16.8 |
| Agree | 26 | 20.8 |
| Strongly Agree | 4 | 3.2 |

**Appendix A.6:** Frequency and Impact of Adverse Effects Experienced by Patients from Pain Treatment Post-Orthopedic Surgery

| **Side Effect** | **Did not experience it at all** | **Did not bother me at all** | **Bothered me a little** | **Moderately bothered me** | **Bothered me a lot** | **Extremely bothered me** |
| --- | --- | --- | --- | --- | --- | --- |
| Unintended weight gain | 40.8% | 17.6% | 21.6% | 12.0% | 6.4% | 1.6% |
| Excessive fatigue | 20.8% | 20.8% | 26.4% | 23.2% | 8.8% | 0.0% |
| Excessive drowsiness | 12.0% | 19.2% | 30.4% | 23.2% | 13.6% | 1.6% |
| Inability to concentrate | 24.0% | 14.4% | 34.4% | 20.0% | 6.4% | 0.8% |
| Nausea | 40.0% | 14.4% | 23.2% | 13.6% | 8.8% | 0.0% |
| Diarrhea | 52.0% | 15.2% | 16.0% | 12.8% | 3.2% | 0.8% |
| Dizziness | 43.2% | 12.8% | 30.4% | 11.2% | 1.6% | 0.8% |
| Constipation | 57.6% | 11.2% | 18.4% | 8.8% | 4.0% | 0.0% |
| Skin rash | 59.2% | 16.8% | 12.8% | 6.4% | 3.2% | 1.6% |
| Stomach pain | 52.8% | 14.4% | 13.6% | 8.8% | 8.0% | 2.4% |
| Stomach burning sensation | 58.4% | 9.6% | 16.0% | 7.2% | 5.6% | 3.2% |

‏

**Appendix A.7:** Patient Satisfaction with Pain Treatment Post-Orthopedic Surgery

| **Pain treatment satisfaction subscale** | **Frequency** | **Percent** |
| --- | --- | --- |
| **The information you received about the pain you are experiencing and its treatment** |  |  |
| Very Dissatisfied | 5 | 4.0 |
| Dissatisfied | 38 | 30.4 |
| Neutral | 22 | 17.6 |
| Satisfied | 52 | 41.6 |
| Very Satisfied | 8 | 6.4 |
| **The amount of time doctors spend with you during their visits/consultations** |  |  |
| Very Dissatisfied | 3 | 2.4 |
| Dissatisfied | 44 | 35.2 |
| Neutral | 30 | 24.0 |
| Satisfied | 37 | 29.6 |
| Very Satisfied | 11 | 8.8 |
| **The care provided by nursing during pain treatment** |  |  |
| Very Dissatisfied | 3 | 2.4 |
| Dissatisfied | 30 | 24.0 |
| Neutral | 28 | 22.4 |
| Satisfied | 44 | 35.2 |
| Very Satisfied | 20 | 16.0 |
| **The form of the pain medication** |  |  |
| Very Dissatisfied | 2 | 1.6 |
| Dissatisfied | 33 | 26.4 |
| Neutral | 28 | 22.4 |
| Satisfied | 49 | 39.2 |
| Very Satisfied | 13 | 10.4 |
| **How often do you take this medication regularly** |  |  |
| Very Dissatisfied | 4 | 3.2 |
| Dissatisfied | 35 | 28.0 |
| Neutral | 31 | 24.8 |
| Satisfied | 40 | 32.0 |
| Very Satisfied | 15 | 12.0 |
| **The amount of medication for pain relief that you have taken** |  |  |
| Very Dissatisfied | 2 | 1.6 |
| Dissatisfied | 31 | 24.8 |
| Neutral | 35 | 28.0 |
| Satisfied | 48 | 38.4 |
| Very Satisfied | 9 | 7.2 |
| **The time it takes for the pain medication to start working** |  |  |
| Very Dissatisfied | 4 | 3.2 |
| Dissatisfied | 35 | 28.0 |
| Neutral | 31 | 24.8 |
| Satisfied | 48 | 38.4 |
| Very Satisfied | 7 | 5.6 |
| **The level or amount of pain relief your medication provides** |  |  |
| Very Dissatisfied | 3 | 2.4 |
| Dissatisfied | 40 | 32.0 |
| Neutral | 19 | 15.2 |
| Satisfied | 55 | 44.0 |
| Very Satisfied | 8 | 6.4 |
| **The duration of pain relief provided by your medication** |  |  |
| Very Dissatisfied | 5 | 4.0 |
| Dissatisfied | 19 | 15.2 |
| Neutral | 41 | 32.8 |
| Satisfied | 52 | 41.6 |
| Very Satisfied | 8 | 6.4 |
| **Variable** | **M** | **SD** |
| Pain treatment satisfaction subscale score | 3.23 | 0.82 |

M, mean; SD, standard deviation.

**Appendix A.8:** Variations in sleep quality based on demographic characteristics

| **Variable** | **N** | **Mean** | **T (F)** | **P value** |
| --- | --- | --- | --- | --- |
| Male  Female | 64  53 | 8.21  8.17 | 0.07 | 0.93 |
| Employment status  Employed  Unemployed | 86  31 | 8.15  8.32 | 0.11 | 0.52 |
| Insurance  Insured  uninsured | 90  27 | 8.32  7.78 | 0.76 | 0.32 |
| Previous surgery  Yes  No | 39  78 | 8.09  8.25 | 0.088 | 0.73 |
| Previous pain medications  Yes  No | 77  40 | 8.39  7.81 | 1.18 | .279 |
| Marital status  single  married  widowed | 68  42  7 | 8.03  8.46  8.19 | 0.32 | 0.12 |
| Educational level  primary  secondary  bachelor  post grad | 27  38  49  3 | 8.30  8.03  8.30  7.53 | 0.21 | 0.31 |
| Orthopedic surgery  joint replacement  spinal surgery  fixations  knee surgery  others | 27  18  28  21  23 | 8.20  8.17  8.44  7.66  8.40 | 0.283 | 0.81 |
| Chronic diseases  none  DM  HTN | 78  14  25 | 8.45  7.20  7.95 | 1.37 | .25 |
| Exercise  none  1-2 times weekly  3-4 times weekly  daily | 70  36  7  4 | 8.11  8.06  8.86  9.62 | .55 | .64 |
